# Financing a sustainable ocean economy

**DOI:** 10.1038/s41467-021-23168-y

**Published:** 2021-06-08

**Authors:** U. Rashid Sumaila, Melissa Walsh, Kelly Hoareau, Anthony Cox, Louise Teh, Patrízia Abdallah, Wisdom Akpalu, Zuzy Anna, Dominique Benzaken, Beatrice Crona, Timothy Fitzgerald, Louise Heaps, Ibrahim Issifu, Katia Karousakis, Glenn Marie Lange, Amanda Leland, Dana Miller, Karen Sack, Durreen Shahnaz, Torsten Thiele, Niels Vestergaard, Nobuyuki Yagi, Junjie Zhang

**Affiliations:** 1 Fisheries Economics Research Unit, University of British Columbia, Vancouver, BC Canada; 2 Marine Conservation Finance Consulting and Ocean Finance Initiative, Asian Development Bank, Metro Manila, Philippines; 3 University of Seychelles James Michel Blue Economy Research Institute, University of Seychelles, Anse Royale, Seychelles; 4 Organization for Economic Co-operation and Development, Paris, France; 5 Universidade Federal do Rio Grande—FURG, Rio Grande, Brazil; 6 Ghana Institute of Management and Public Administration, Accra, Ghana; 7 SDGs Center Universitas Padjadjaran, Kota Bandung, Indonesia; 8 Australian National Centre for Ocean Resources and Security, Wollongong, NSW Australia; 9 The Royal Swedish Academy of Science, Stockholm, Sweden; 10 Environmental Defense Fund, Washington, DC USA; 11 WWF-United Kingdom, Surrey, UK; 12 The World Bank, Washington, DC USA; 13 Oceana-Europe, London, UK; 14 Ocean Unite, Washington, DC USA; 15 Impact Investment Exchange (IIX), Singapore, Singapore; 16 London School of Economics, London, UK; 17 University of Southern Denmark, Odense, Denmark; 18 University of Tokyo, Tokyo, Japan; 19 Duke Kunshan University, Kunshan, China

**Keywords:** Environmental social sciences, Environmental economics

## Abstract

The ocean, which regulates climate and supports vital ecosystem services, is crucial to our Earth system and livelihoods. Yet, it is threatened by anthropogenic pressures and climate change. A healthy ocean that supports a sustainable ocean economy requires adequate financing vehicles that generate, invest, align, and account for financial capital to achieve sustained ocean health and governance. However, the current finance gap is large; we identify key barriers to financing a sustainable ocean economy and suggest how to mitigate them, to incentivize the kind of public and private investments needed for topnotch science and management in support of a sustainable ocean economy.

## Abstract (web summary)

The ocean supports many livelihoods, but this is currently not sustainable with pressures on the climate and ecosystems. Here, in this perspective, the authors outline the barriers and solutions for financing a sustainable ocean economy.

## Introduction

### A sustainable ocean economy

Covering over 70% of Earth’s surface, oceans are a natural asset, which, along with soils and forests, make up the world’s stock of natural capital. These natural assets generate vital ecosystem goods and services, such as food, climate regulation, coastal protection, and cultural value that support planetary life and human survival and well-being worldwide^1–4^. Oceans contain a diversity of renewable and non-renewable resources (e.g. fisheries, oil, and gas deposits) that provide the intermediate inputs, such as waves and fish stocks, to support ocean-based industries like renewable energy and seafood production. Here we consider the sum of economic activities of ocean-based industries and the assets, goods, and services generated by marine ecosystems to constitute the ocean economy^5^.

The size of the global ocean economy, which includes fishing, shipping, offshore wind, maritime and coastal tourism, and marine biotechnology, was estimated at USD 1.5 trillion, or 2.5% of global gross value added in 2010. This value is growing rapidly, and prior to the COVID-19 pandemic, it was projected to increase to USD 3.0 trillion in 2030^5^. Note that this is likely an underestimate since many valuations do not include benefits that lack a market value, such as cultural, social, and ceremonial values. Another study conducted in 2015 estimated the annual gross marine product value to be at least USD 2.5 trillion per year, based on the value of the ocean’s total asset base consisting of the direct output of the ocean from marine fisheries and coastal ecosystems, marine trade and transport, and adjacent assets (e.g. tourism and carbon absorption)^6^. The ocean economic sectors with the strongest growth potential include marine aquaculture, fish processing, offshore wind, and shipbuilding^5^.

A vibrant ocean economy depends on sustainable and healthy oceans. However, many aspects of current ocean resource use patterns make it unsustainable. Human transformation of marine ecosystems has resulted in widespread biodiversity loss and habitat damage. This is not a new phenomenon; for instance, intense exploitation of Caribbean coral reefs in the 17–19th century led to massive losses in large vertebrates, fish, and sharks^7^. Currently, overfishing, destructive fishing practices, direct habitat damage, and pollution are major anthropogenic threats to the future sustainability of oceans and their resources^8–11^. Moreover, some components of the ocean economy, such as deep-sea mining, are inherently damaging to the ocean environment^6^. In particular, climate change is driving unprecedented changes that severely affect ocean health, and the ocean’s ability to sustain the flow of ecosystem goods and services upon which human and societal well-being depend^9,11^.

The 2019 IPCC Report on the Ocean and Cryosphere^10^ and the Blue Paper on climate change^11^ both report significant impacts of climate change on the ocean economy. According to the IPCC, the ocean is projected to transition to unprecedented conditions over the 21st century due to increased temperatures, greater upper ocean stratification, acidification, deoxygenation, altered net primary production, and more frequent marine heat-waves, El Niño, and La Niña events. Although the rates and magnitudes of these changes are expected to be smaller under low greenhouse gas emission scenarios, they will nonetheless have an impact on marine ecosystems and species. For all emission scenarios, there is a projected decrease in the biomass of marine animal communities, fisheries catch potential, and a shift in species composition over the 21st century. These projected decreases will affect the global ocean economy through impacts on livelihoods, food security, and income of marine resource dependent communities, and is expected to be worse in the tropics. Further, the ocean’s role in supporting cultural, recreational, and intrinsic values for human well-being is likely to be diminished due to the projected long-term loss and degradation of marine ecosystems. Similarly, the latest IPBES report emphasizes that nature and nature’s contribution to people (e.g. ecosystem goods and services), including those from the ocean, are deteriorating worldwide. For instance, 66% of the world’s ocean area is experiencing increasing cumulative impacts^4^.

Clearly, there is a need to change existing practices to ensure that they are compatible with an ocean economy that is sustainable. While there is no consensus yet on a globally accepted definition of a Sustainable Ocean Economy (SOE), we adopt the definition provided by the High Level Panel on a SOE: “Development of the ocean economy in a way that balances the needs of people, planet, and prosperity”, which is amplified in Blue Paper 14 on Integrated Ocean Management^12^ as one that ensures “long-term, sustainable use of ocean resources in ways that preserve the health and resilience of marine ecosystems and improve livelihoods and jobs, balancing protection and prosperity”.

This paper focuses on the role that ocean finance can play in supporting sustainable development of the ocean economy. Finance plays a pivotal role in advancing a SOE because funds are needed for investing in governance and initiatives to promote sustainable ocean use while reducing threats and mitigating the underlying drivers of ocean health decline. The objective of this paper is thus to gain a better understanding of the state of ocean finance within the context of enabling a SOE by identifying current barriers to sustainable ocean finance, and how to address them.

### Ocean finance

Ocean finance deals with the demand for, and supply of financial capital for investing in ocean-related economic activities and governance. For the ocean economy to be sustainable, ocean finance has to be adequate and directed toward sustainable use and governance of the ocean and its resources. Key elements of financing a SOE include generating, investing, aligning, and accounting for financial capital^13^. This encompasses local, national, and international level financial instruments that are provided by, and/or accessed by individuals, public and private companies, governments, and other non-governmental/inter-governmental institutions.

Financial capital can be used in diverse ways for advancing a SOE. Companies may use capital to finance development of more sustainable products, technology, and gain access to new sustainability friendly-markets. Governments and non-governmental organizations (NGOs) may use funds for implementing conservation policies or investing in strengthening the enabling environment for the private sector to finance and insure sustainable ocean economic activities. Individuals can invest in public equity, i.e. buying shares in publicly traded companies that partake in environmental standards and principles. The more established ocean economic sectors e.g. shipping, tourism, industrial fishing, and energy, can and are often publicly traded to raise funds.

**Fig. 1:**
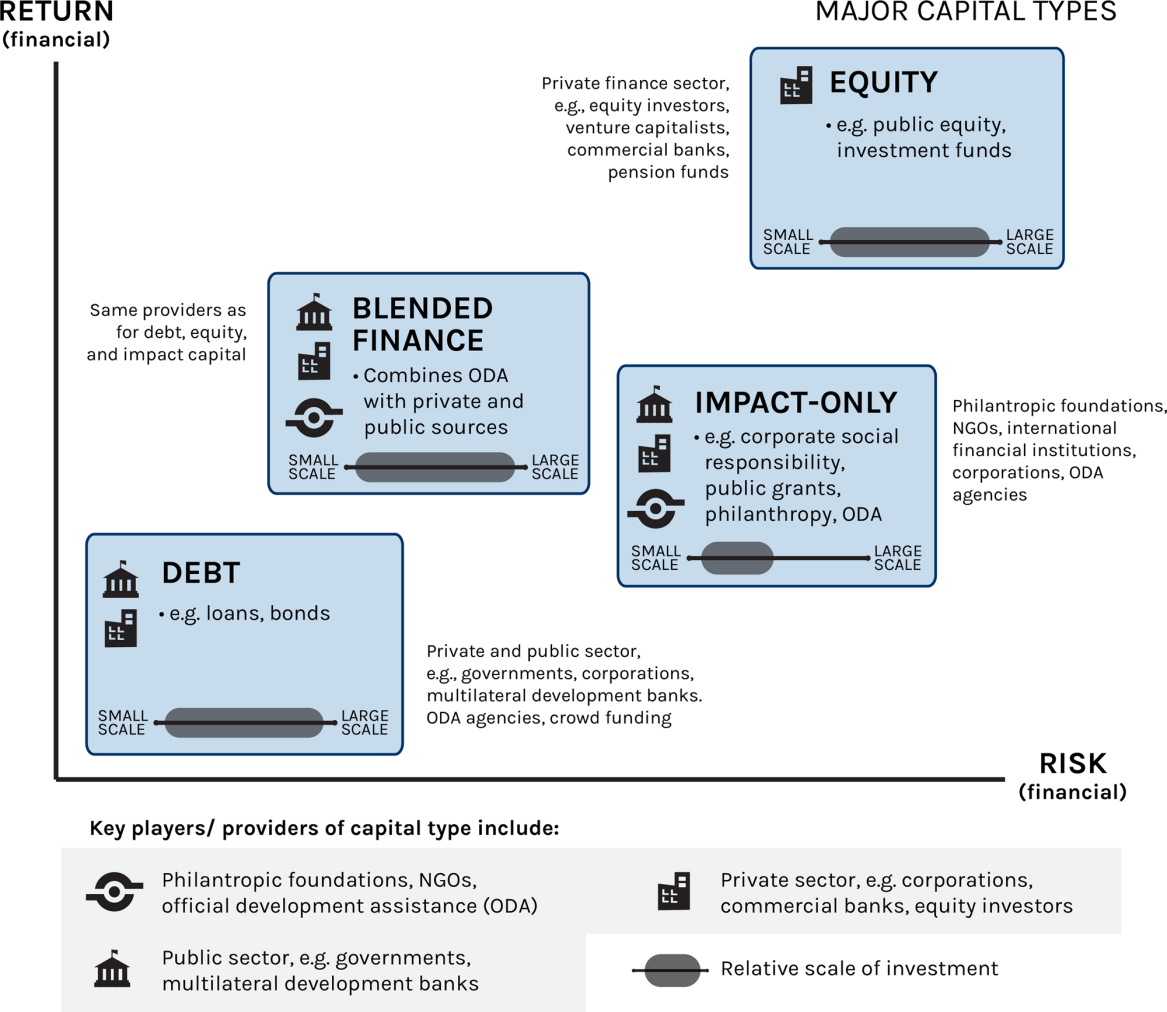
Characterization of major ocean finance capital types. Summary of major capital types, the level of risk vs. return for each capital type, and the key providers of these capital^14^.

Financial instruments used to either finance a SOE, or as a basis for generating new financial capital for promoting sustainable ocean resource use include traditional loans and grants, carbon markets, and insurance instruments (Fig. 1). The deployment of these different capital types depends on the expected returns from the investment, which in turn depends on the risk-return equations faced by investors (Fig. 1).

Ocean finance can play a vital role in supporting sustainable development of the ocean economy by directing investments to activities, policies and actions that minimize ocean risks and maximize social equity and environmental sustainability. Some of the needed investments in a SOE are likely to generate competitive market returns and thus able to attract private finance, whereas other investments are capable of generating positive but below market returns. For these investments to be attractive to the private sector, some form of public or philanthropic co-financing or blended finance (Fig. 1) would be needed. Finally, investments that are needed to support certain important ecosystem functions but are incapable of generating any market return (Table 1) are unlikely to attract private finance. In this case investments would have to be paid for through public and/or philanthropic sources (Fig. 1).

**Table 1:** Summary of major capital types and key providers of these capital^3^.

Capital type	Description	Key players/providers
Impact-only		
Corpoate Social Responsibility investment	This is usually long term, but small scale in comparison to larger types of commercial finance. Expected rates of return are usually below market rates	Philantropic foundations, NGOs, international financial institutions, corporations, official development assistance (ODA) agencies
Public grants		
Philanthropic grants		
Public financing		
Official development assistance		
Debt		
Loans	Low-risk, low-reward types of capital that offer low or market rates of return. They are variable in scale, ranging from micro-finance to large-scale corporate loans	Private and public sector, e.g. governments, corporations, multilateral development banks. ODA agencies, crowd funding
Bonds		
Equity		
Public equity	Equity involves taking an ownership stake in an investment. Some types of equity are high risk, high-reward and can offer greater than market return. The scale of equity is very variable, ranging from micro-finance to multi-million dollar investments	Private finance secctor, e.g. equity investors, venture capitalists, commercial banks, pension funds
Equity investment (investment funds)		
Blended finance	Combines official development assistance with other private or public resources, in order to ‘leverage’ additional funds from other actors. It generally provides below market rates of return	Same providers as for debt, equity, and impact capital
Subsidies	Financial aid, commonly provided by governments, to an economic sector in order to promote economic and social policy	Public sector (governments)

**Fig. 2:**
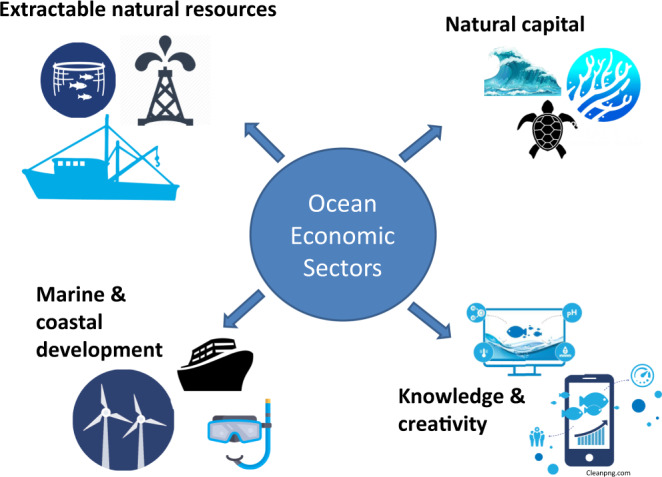
Ocean economic sectors. Depiction of the four broad economic sectors that make up the ocean economy: extractable natural resources, natural capital, marine and coastal development, and knowledge and creativity.

Financing a SOE involves investments in the various mature and emerging sectors that make up the ocean economy (Table 2). Box 1 and Fig. 2 summarize examples of investments in four ocean economic sectors that partly follow the categorization used by Vos and Hart^14^: natural capital, extractable marine resources, marine and coastal development, and knowledge and creative sectors.

**Table 2:** Examples of established and emerging sectors in the ocean economy.

Established sectors	Emerging sectors
Capture fisheries	Marine aquaculture
Seafood processing	Deep and ultra-deep-water oil and gas
Shipping	Offshore wind energy
Ports	Ocean renewable energy
Shipbuilding and repair	Marine and seabed mining
Offshore oil and gas	Maritime safety and surveillance
Marine manufacturing and construction	Marine biotechnology
Maritime and coastal tourism	High-tech marine products and services
Marine business services	
Marine research and development and education	
Dredging	

Source: OECD^1^.

Box 1: Examples and illustration about the type and scale of investment in each of the four ocean economic sectors.

**Table:** 

Ocean sector type	Example investments
Natural capital: development and investment flows are directed to the natural assets that underpin ecosystem services, e.g. conservation and restoration of natural systems, and do not involve the creation of built structures. Perceived investment risks in this sector are relatively high as natural capital is not a conventional invesment	Natural infrastructure: Proposed restoration of Louisiana wetland ecosystems for flood defence (Project Scale: <US$100 million)^78^.
Extractable natural resources: involve human activities that remove or produce a physical good from the ocean. These sectors, such as fisheries, have received extensive investment for many years. As such, sustainable development of these sectors involve redirecting existing investment towards sustainable pathways, while simultaneously generating new sources of capital.	Wild-caught marine fisheries (small scale): Development of aggregated fish processing site, sourcing from multiple small-scale fisheries (Project scale: <US$ 1 million)^79^. Sustainable aquaculture: Indian Ocean trepang fish farm expansion (Project Scale: <US$10 million)^80^. Marine bioprospecting: Early-stage investment in bioprospecting firm (Project Scale: <US$10 million)^81^.
Marine & coastal development: creation of new, fixed, physical assets at sea and along the coast. These include sectors, such as shipping, that occur physically on the ocean (e.g. maritime transportation, ocean-based renewable energy), or land-based sectors that have a clear marine impact, e.g. marine ecotourism, port, harbour, and boat construction, waste management. Sectors such as shiping have been invested in for many years, and will require redirecting capital towards sustainability, whereasnewer sectors such as marine ecotourism will need new sources of capital.	Nature-based infrastructure: Public investment in nature-based generation of new beaches, North Sea coast (Project Scale: <US$100 m)^82^. Maritime transportation: Fleet-wide vessel retrofitting for fuel efficiency and lower emissions (Project Scale: >US$100 million)^83^.
Knowledge and creative sector: includes academic, non-academic, professional, and public sector services that conduct research and development activities to create new knowledge and innovation for a sustainable ocean economy. Some of these emerging sectors, e.g. ocean technology development, may be perceived as high risk, and require new sources of high-risk high-reward capital.	Academic research: Ongoing establishment of a new Blue economy Institute at the University of Nairobi (Project Scale: >US$100 million).

### Rationale for financing a sustainable ocean economy

Unsustainable use of oceans and their resources has led to the depletion of fish stocks and biodiversity, and increased pollution and habitat damage, among other negative impacts; cumulatively, these impacts reduce ecosystem resilience and increase humans’ vulnerability to future global change^10,15,16^, thereby incurring a huge economic cost to society. The costs of inaction towards the conservation and sustainable use of the ocean are high. The total estimated cost of coastal protection, relocation of people and loss of land to sea-level rise is projected to range from USD 200 billion to a trillion USD annually by 2100, depending on the increase in sea level (0.5–1 m)^17,18^. Already, it appears that a one metre mean sea-level rise is likely in 2100 under a RCP8.5 scenario^10^. Noone et al.^8^ state that in the absence of proactive measures to mitigate climate change, the cost of climate impacts on the ocean could be an additional USD 322 billion a year by 2050; this cost includes losses in fisheries, tourism, and ocean carbon absorption, damages arising from sea-level rise and storms.

**Fig. 3:**
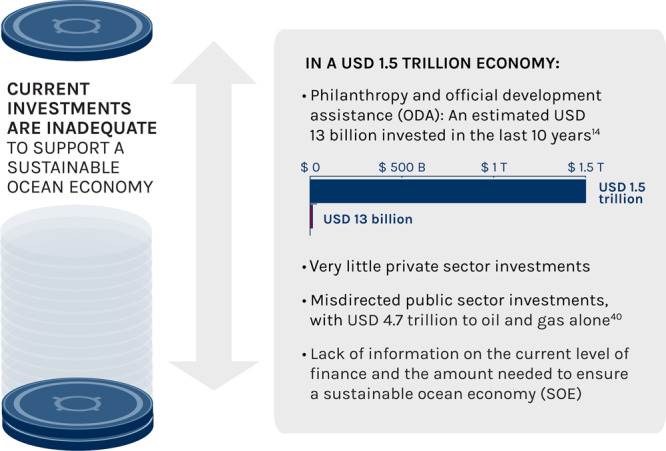
Ocean finance gap. Current investments are insufficient to support a sustainable ocean economy, resulting in a large ocean finance gap.

Despite the huge costs of inaction, public and private investments in the ocean economy are insufficient, resulting in significant financing gaps (Fig. 3). For example, Marine Protected Areas (MPAs), areas of the ocean that are completely or partially closed to human activities, are a natural capital sector that form an important component of a SOE because of their role in promoting species and ecosystem protection and recovery^19–21^. However, in the Mediterranean alone, Binet et al.^22^ estimated that there was a financing gap of USD 776.4 million per year for effective management of MPAs. Globally, the total cost of establishing and maintaining MPAs in 2018 was estimated at USD 2.3 billion^23^. Currently, only 2.3% of declared MPA areas are Highly or Fully Protected^24^. Using this information together with reported subsidies data^23^, we estimate that to get to the 10% target of Highly or Fully Protected areas would require at least USD 7.7 billion globally. Libes and Eldridge^25^ report that Sustainable Development Goal (SDG) 14—Life Below Water—currently receives the lowest impact investment of all SDGs. Similarly, blended finance vehicles (i.e. combinations of capital from different providers, e.g. public-private partnerships, in order to provide larger amounts of capital for projects that may be considered too risky for any one capital provider on their own) presently directed to SDG 14 are the least across all SDGs^26^, pointing to the need for stronger efforts in increasing the quality, quantity and impact of ocean finance. The “business as usual” trajectory entails great risk to ocean users, including the future well-being of hundreds of millions of people in coastal and island communities. This runs counter to the 2030 Agenda for Sustainable Development and all seventeen SDGs, in particular, SDG 14 and those focusing on hunger (SDG 2), poverty (SDG 1), work and economic growth (SDG 8), and climate action (SDG 13).

One study estimated that the gap in conservation financing for all ecosystems, which includes funds for a SOE, was USD 300 billion globally^27^. Though the proportion of this amount that is relevant to the ocean has not been identified, the ocean financing gap is likely very high; it is estimated that to meet the global need for conservation funding in general, investable cash flows from conservation projects need to be at least 20–30 times greater than they are today^27^. Sumaila et al.^28^ report that currently ∼0.002% of global GDP is invested in the conservation and sustainable use of biodiversity generally, and that about four times the current level of investment is required to meet conservation needs. It is evident that current investment for a SOE is insufficient. While an estimated USD 8 billion from philanthropy and USD 5 billion from Official Development Assistance (ODA) were invested in sustainable development of the ocean economy in the past 10 years (Fig. 3), this level is insufficient to drive the change needed to achieve a SOE^14^.

## Barriers to financing a sustainable ocean economy

**Fig. 4:**
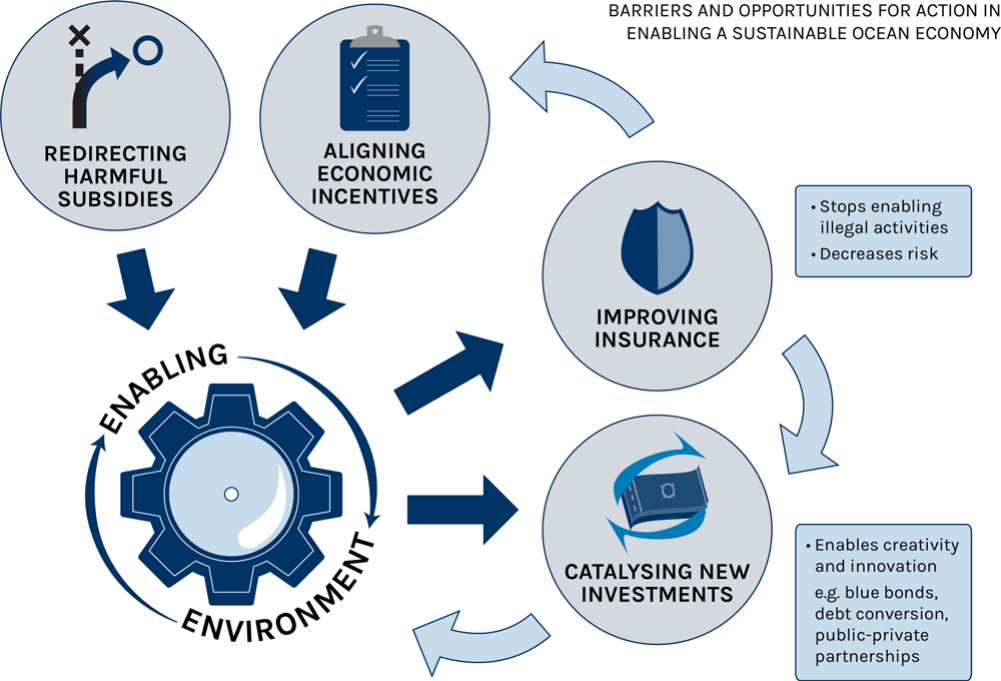
Creating an enabling environment for ocean finance. Barriers and options for action in support of sustainable ocean finance.

We outline three key barriers that are currently preventing the flow of adequate ocean finance. The first barrier we consider is the set of challenges we categorize under enabling environment, i.e. an environment conducive to attracting sustainable ocean finance. Public policies such as those pertaining to the provision of subsidies and how they help align incentives are crucial to creating such an enabling environment (Fig. 4). We further categorise two other barriers to financing a SOE as finance and investment, and insurance and risk mitigation.

### Enabling environment

Effective and stable regulatory and policy environments to attract investment and funding are lacking. Current financing conditions are not attractive enough to encourage investors and financiers to invest sustainably in the ocean economy. In particular, current policies and regulations that strengthen the sustainable management of natural capital, and that facilitate and incentivize social enterprise and new forms of capital are insufficient for attracting the needed quantity and quality investment to the ocean economy.

Information and knowledge about the ocean and its economic, social, and environmental value is missing or inadequate. For appropriate and adequate finance to flow into the ocean economy, its overall contribution to the ocean economy needs to be understood and measured more comprehensively than it currently is. Even in cases where ocean finance data are available, they are often too aggregated in existing national accounts^29^. The good news is that gaps in knowledge about the ocean’s significant economic contribution are beginning to be published^5,6^. For instance, Hoegh-Guldberg et al.’s^6^ estimate of USD 2.5 trillion per year in gross marine product would, if the ocean were a country, rank it seventh in the world in terms of GDP. As this study is several years old, it is likely that the contribution of the ocean economy is now higher. In addition, many studies do not capture the ocean’s full spectrum of values arising from multiple ecosystem services that are generally not reflected in market prices^30^. These include benefits such as natural hazard protection, carbon sequestration, climate mitigation, and pollution buffering^31^. The values of these services can be very high. For instance, carbon sequestration in the Mediterranean Sea was estimated to range between 100 and 1500 million € per year^32^.

Market distortions are undermining a SOE (Fig. 4). Ocean economic activities that generate negative externalities such as fossil energy extraction, unsustainable fishing and aquaculture, and non-green shipping^33^ receive subsidies. The International Monetary Fund estimates that 6.3% of global GDP (USD 4.7 trillion) was provided as fossil fuel subsidies in 2015 to both ocean and land-based businesses^34^, and USD 35 billion in subsidies is allocated to global marine fisheries each year, of which USD 22 billion is allotted to harmful subsidies that lead to overcapacity and overfishing^23^. The OECD estimates that on average, governments of member countries spend up to 20% of the value of fisheries landings in support of the sector, amounting to USD 7 billion per year. A large percentage of subsidies are currently allocated to large-scale industrial operations^35^, which can make small-scale enterprises less economically viable^36–38^.

Beneficiaries and impactors of the ocean do not consistently nor adequately pay for access, use, or management of ocean resources. Maritime countries are generating large economic outputs from the ocean economy. For instance, the ocean economy accounts for 15–20% of GDP in East Asian countries^39^, while in Mauritius, the ocean economy accounts for over 10% of GDP^40^. However, the cost of ocean management is currently not being borne by those exploiting it, including direct harvesters and consumers. Consequently, there is underfunding of ocean governance and the health that underlies the ocean’s economic outputs. The private sector also benefits from, and impacts upon the ocean, but generally does not contribute sufficiently to the management of the ocean economy.

A framework for guiding SOE investments, and a taxonomy of SOE investments are not universally defined. A framework and taxonomy to guide which investments are supportive of a SOE, i.e. “blue” investments have not yet been universally adopted. It is necessary to provide a classification system of activities that comply with the principles of a SOE, thereby guiding investment decisions and development policy towards a SOE. An example is the UNEP (United Nations Environmental Programme) Sustainable Blue Economy Finance Principles^41^ whereby UNEP works with financial institutions to incorporate environmental, social, and governance issues into business principles, and to integrate sustainability principles into financial market practices. While efforts towards common frameworks and taxonomies are underway, e.g. by the Asian Development Bank^42^), the current lack of such a framework and taxonomy results in less investments and development policies towards a SOE than could be possible.

### Finance and investment

There is a lack of high quality, investible projects with appropriate deal size and risk-return ratios to match available capital^43^. While there is no shortage of investment capital available globally, the immediate lack of high quality, investible projects that would contribute to a SOE is a substantial challenge^44–47^. Many ocean interventions require grant capital that generate very low, or no financial returns at all^14^.

For the minority of projects that do benefit the ocean and generate a financial return, many are (1) too small to be financially viable once the costs of due diligence are considered; and (2) too high in risk-return profile due to the relatively more unpredictable conditions that ocean economic sectors operate under compared to those on land. Nevertheless, there are new innovative projects which have overcome these barriers. For instance, Swimsol, a European based company, set up the first floating solar panels in the Maldives. It achieves a 3–8% rate of return from its investment by engaging in a long-term power purchasing agreement with its client, usually a hotel or utility company. Both parties benefit from this agreement as Swimsol’s solar power is 10–50% cheaper compared to its client’s current power generation costs, which is based on diesel generators (pers comm from D. Schmitz of Swimsol).

Access to ocean finance and resources is limited and not equitably distributed. Ocean resources are “rarely equitably distributed”, and many of their benefits are captured by a few^38,48^. At the same time, a large proportion of the costs from ocean-based economic activities are borne by women, youth, and marginalized communities. This inequity is exemplified by the provision of subsidies to the fossil fuel sector, wherein subsidies to big business only serve to increase inequality, which ultimately leads to unfair distribution of ocean economic values and benefits to small-scale actors^38^. Given the important food security, livelihoods and cultural roles that small-scale ocean economic activities play worldwide^38^, the last thing public policies should do is to disadvantage them if we want to meet the SDGs, especially, SDGs 1- 5, and 10 (No Poverty; Zero Hunger; Good Health and Well-being; Quality Education; Gender Equality; Reduced Inequality).

### Insurance and risk mitigation

Ocean investments often have high risks but the enabling regulatory environment for attracting investors is not in place. Overcoming the higher risk profile associated with the ocean sector will require addressing a number of challenging enabling conditions in order to attract investments and new forms of finance. These challenges include human capacity constraints, data challenges and higher risk of operation (see 2.1 above). In addition, structural challenges related to the ocean make scale and replication more complex than in more familiar terrestrial sectors (notably related to tenure and ownership, monitoring, and enforcement). In order to attract large-scale investments, it is critical to find ways to de-risk the enabling environment associated with ocean-based sustainable development projects and activities. While marine insurance is a strategy for managing commercial risks for shipping, aquaculture, fishing, and other offshore industrial activities, it does not cover all risks to the ocean economy (e.g. blue carbon and nature-based infrastructure investments). Further, smaller sized companies may not always purchase insurance, partly due to lack of affordability and availability^49^, implying that other strategies besides insurance are needed for mitigating risk.

## Addressing the barriers to financing a SOE

Despite the barriers outlined in “Barriers to financing a sustainable ocean economy”, there are numerous actions, which we outline below, that can be taken to address the challenge of financing a SOE.

### Enabling environment

**Fig. 5:**
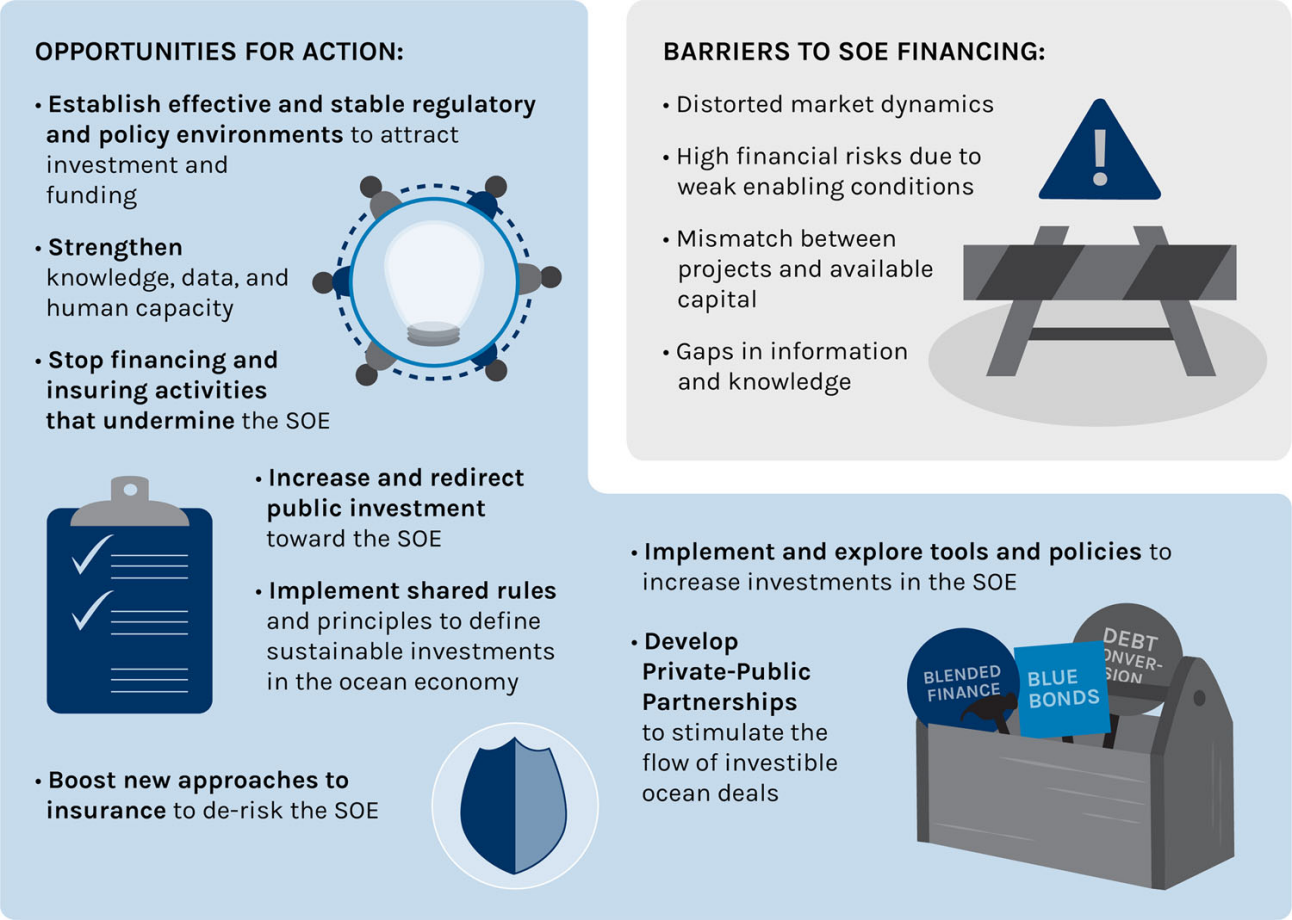
Actions that create barriers to, and opportunities for, a sustainable ocean economy. Key barriers to the flow of finance to the ocean economy and opportunities for action to remove them.

Establish effective and stable regulatory and policy environments to attract investment and funding. Governments and multilateral agencies can create attractive financing conditions (Fig. 5). This can be achieved by reforming policies and creating regulations that strengthen the sustainable management of natural capital and that facilitate and incentivize social enterprise and new forms of capital^50,51^. This might include national policies that secure tenure and establish robust enforcement mechanisms in the fishing sector^52–54^, or that support technology transfer and incentivize renewable energy^55,56^. Governments should also create conditions that provide access to financing, savings, micro-insurance, and other services^57^. Market-based incentives, such as certification, can increase the investment potential for projects by providing some assurances on sustainability throughout the supply chain and implementing more transparent monitoring approaches^58^. This is needed because the low traceability in many marine seafood supply chains, for e.g. impedes the capacity of corporate investors to steer investments towards more sustainable practices^59,60^. Governments can create stronger incentives for certification, while the conservation and development sectors will need to provide technical assistance.

Strengthen national, regional, and global data infrastructure to increase transparency, grow knowledge, and build effective human capital worldwide, particularly in developing countries. More consistent and comprehensive monitoring and reporting on finance for a SOE, especially financial flows for biodiversity^61^ across both the public and private sectors is needed. The UN System for Environmental Economic Accounting, which provides a standardized framework to account for environmental protection and management expenditure in a manner that is interoperable, is a good effort that needs to be expanded to include ocean finance information more comprehensively^29^. Similarly, the recently established Global Ocean Accounts Partnership has yet to include ocean financial flows in its framework for ocean accounting and capacity-building activities^62^. Countries should, individually and regionally, invest in data and analysis. Government budgets need to be able to track spending on ocean-based sustainable development. Developing ocean data architecture at sufficient granularity will help private investors to get sufficient information to make key investment decisions, and support good business plans and practices. To build the kind of information needed to attract investments into the ocean economy, we need to significantly increase the human capacity for acquiring, investing, and aligning ocean finance in many developing maritime countries. Effective capacity building in the areas of ocean finance, insurance, and the application of fiscal instruments, especially from multilateral organizations or bilateral aid, are needed urgently to support investment for a SOE.

Correct market distortions through taxation, pricing services and the re-purposing of harmful subsidies to more sustainable and equitable uses. Fiscal (e.g. subsidies, fees, and taxes) and non-fiscal (e.g. tradable permits and social norms) incentives should be deployed to ensure that the effects of negative externalities are eliminated while those of positive externalities are promoted. Importantly, current funding of unsustainable ocean use needs to end, and the saved funds redirected to sustainable use. Environmentally or socially harmful subsidies could be diverted to support the move to renewable energy or related sectors. Redirecting harmful subsidies to beneficial uses is an opportunity to catalyze a SOE, and improve gender and other equity issues^38^. International negotiations and mandates, such as Asia-Pacific Economic Cooperation (APEC), the G-20, SDG 14.6, WTO negotiations and the G7, have called repeatedly for the phasing out of inefficient fuel subsidies and distortive support measures^63^. This momentum for reform can be channeled into better policies for a SOE.

Allocate a greater proportion of ocean economic output into multi-sector ocean governance strategies. A resilient ocean economy requires rigorous and comprehensive ocean governance, and hence needs continuous funding. Diverting even a small amount of the ocean’s projected gross value added of USD 3.0 trillion in 2030 could raise substantial funds to strengthen ocean governance and put in place the necessary pre-requisites to attract investment into the sector. Countries could capture these revenues using a combination of domestic taxes, levies, fines, fees, and other mechanisms that monetize ocean benefits and ocean impacts, such as payments for ecosystem services. These mechanisms, in combination with proper management measures—which may include assigning access rights to indigenous coastal communities, or limiting access to ocean resources—can generate revenues to help bring about a SOE. Funds could also be allocated to multi-sector ocean governance strategies and marine spatial plans, including management of all significant threats and impacts to ocean health as determined by a country.

Environmental Fiscal Reform refers to a range of taxation and pricing measures that can raise revenues while furthering environmental goals^64^. Such mechanisms provide an opportunity to align public and private incentives in the ocean economy, and are also a mechanism to share the wealth of ocean resources more broadly in society. Auctions for access to ocean resources can help measure their value and generate funds to use for sustainable management, and for the benefits of communities at large. The vessel day scheme of the Parties to the Nauru Agreement provides an example whereby pooling of vessels days are auctioned to distant fishing nations. This ensures the owners receive the full value of these ocean resources from users. Auctions need to consider community, customary, and Indigenous rights, such as by reserving quotas for Indigenous or local fishing communities, the establishment of license banks and funding mechanisms for community-ocean business associations^65^. ODA can be an important source of funding for the conservation and sustainable use of the ocean, especially in the poorest and most vulnerable countries. Initial estimates reveal that ODA-related ocean funding remains limited and that much scope exists to test and further develop financial instruments that use ODA innovatively to mobilize additional resources, such as blue bonds and other blended finance arrangements.

Develop and encourage universal adoption of a common framework and taxonomy to define sustainable investments into the ocean economy. To guide investment decisions and development policy towards a SOE, it is critical that effective guardrails and guidelines are in place and widely adopted. An essential element of this emerging SOE finance ecosystem will also be the creation of an ocean-based finance taxonomy, i.e. creating a classification system of those activities considered to comply with strong principles for a SOE. The European Commission’s Sustainable Blue Economy Finance Principles is an example of scientifically and economically sound principles that are very high level and therefore relevant across many contexts. However due to the high-level nature of the principles, more detailed guidance alongside a common blue taxonomy is still required.

### Finance and investment

Introduce new financing mechanisms and tools. New financing tools and access to capital markets are needed to act as a positive incentive for sustainable, inclusive and climate resilient ocean activities. Innovative financial instruments (Fig. 5) can enable new entrants, including women, youth, and marginalized communities, into the SOE while reducing the overexploitation of ocean resources. These tools can also facilitate effective management and governance while promoting the security of the ocean space in a context of increased access to new ocean resources. For instance, the IIX Sustainability Bonds (https://iixglobal.com/iix-sustainability-bonds/) are debt securities that can be listed on a social stock exchange; these bonds explicitly target the inclusion of women in economic activities.

Use Green/Blue/Climate bonds that meet investment criteria and accountability requirements (for e.g. Green Bond Principles, Environment, Social, and Governance criteria) and certification to qualify for such labels and ensure the integrity of markets in the investment community. The Climate Bonds Initiative (www.climatebonds.net) has a number of sector criteria, including for marine energy and water utilities. Other relevant initiatives include the Blue Natural Capital Positive Impacts Framework (https://bluenaturalcapital.org) and the technical guideline for blue bonds^66^. At the national level, the Netherlands provides special green investment funds that are exempt from income tax, thus allowing investors in green projects, e.g. green shipping, to contract loans at reduced interest rates (usually ~2% below commercial rates)^67^. These Dutch green funds have already attracted more investment than can be utilized in the available schemes—an encouraging sign for the future prospects of such instruments.

Develop Private-Public Partnerships to stimulate the flow of investible ocean deals needed to overcome the initial short-term capital costs required for investments in projects, such as renewable ocean energy, ocean infrastructure, and rebuilding fish stocks. For developing countries, debt conversion or restructuring programs allow debt owed to creditors to be restructured and converted into agreed upon initiatives that address, for instance, marine conservation and climate change. The debtors are then obligated to execute the initiatives (see Box 2 for an example).

Financial institutions incorporate environmental and social sustainability into their risk assessment and investment frameworks. With their considerable clout, banks (e.g. multilateral and national development banks) can use mechanisms such as corporate debt and covenants^68^ to set and promote a sustainability agenda in all ocean sectors. For instance, initiatives such as the Principles for Investment in Sustainable Wild-Caught Fisheries (www.fisheriesprinciples.org) and the Ocean Disclosure Project (www.oceandisclosureproject.org) can be further developed and applied to other ocean economic sectors. The Poseidon Principles, which already applies to 30% of shipping loans, establishes a framework for assessing and disclosing the climate alignment of ship finance portfolios. They set a benchmark for what it means to be a responsible bank in the maritime sector and provide actionable guidance on how to achieve this (https://www.poseidonprinciples.org/#about). These Principles also establish a common, global baseline to quantitatively assess and disclose whether the lending portfolios of financial institutions are in line with adopted climate goals.

Pension funds, sovereign wealth funds, and large passive investors need to be partners in the effort to achieve a SOE. They can use their clout as significant investors in many extractive sectors^69^. The ‘blockholding’ power that this affords institutional investors gives them significant leverage to influence corporate policy on issues ranging from sustainability policies to corporate governance^70^. The Ocean Sustainability Expectations document issued by Norway’s Norges Bank^71^, which manages one of the world’s largest Sovereign wealth funds, sets out how boards of companies operating within ocean sectors should consider the social and environmental consequences of their business activities on ocean sustainability.

Box 2: Seychelles conservation and climate adaptation trust. The Government of the Seychelles entered into a debt conversion program with the Paris Club (a group of 22 creditor countries), with the assistance of The Nature Conservancy. One of the conditions linked to the debt conversion was the development of the Seychelles Marine Spatial Plan. A new Act was also passed to create the Seychelles Conservation and Climate Adaptation Trust (SeyCCAT) in 2015, which provides a well governed funding mechanism (USD 700,000 in competitive grants per year) for long-term financing of activities relating to the stewardship of Seychelles' ocean resources and blue economy. This has enabled the completion of projects that range from improving knowledge about the Seychelles artisanal fishery, to assessing the effectiveness of a Marine Park in protecting lemon sharks. We recommend that additional debt conversions be designed and implemented to support developing countries to implement ocean governance.

### Insurance and risk mitigation

Boost new approaches to insurance. The insurance industry has the potential to play three important roles as risk managers, risk carriers, and investors. As risk managers, insurers can communicate recommendations for more sustainable practices by their clients and within the communities they serve. As risk carriers, insurers can choose to exclude or restrict access to insurance to clients or projects that engage in unsustainable or illegal practices^72–74^. For instance, an industry-wide statement against IUU (illegal, unreported, and unregulated) fishing, developed by the environmental non-profit organization Oceana, and UNEP’s PSI Initiative was launched in 2017, confirming the commitment of insurers, brokers, and agents to not knowingly insure or facilitate the insuring of IUU fishing vessels^75^. To date, over 30 insurers, insurance market bodies and key stakeholders from five continents have signed and supported the Statement, including some of the largest companies in the world. Finally, insurers are also major institutional investors, and in this role, they can elect to support only those clients or projects that contribute to a SOE, and divest from those that do not. There is also an opportunity for all levels of government—local, national or international—to work with the insurance industry to promote the development of a SOE. At the local level, this could involve making improvements in risk modeling, and at national or international levels, policy and regulatory frameworks could be reshaped to incentivize responsible and sustainable maritime industry practices^76,77^.

Develop and deploy parametric insurance instruments (a type of insurance coverage that pays out an agreed upon sum based on the expected loss arising from a trigger event) in support of ocean health. Examples include: (i) parametric cyclone insurance for a segment of the Great Sea Reef in Fiji to incentivize preparedness and finance rapid response and early recovery after major cyclone shock events; (ii) insurance for marine thermal shock events using a sea surface temperature index, to help mitigate the economic consequences of tourism revenue decline due to sudden natural asset degradation in Palau; and (iii) livelihood protection as a social benefit through parametric insurance to support fishers’ resilience and incentivize improved fisheries management in Vanuatu. By risk-adjusting these types of investments, it is possible to develop, replicate, and scale these types of examples around the world. The Ocean Risk and Resilience Action Alliance is designed to help drive the development of such innovative finance products to regenerate coastal natural capital and build resilience in the most exposed and vulnerable regions and communities of the world.

## Concluding remarks

A healthy ocean that supports a SOE requires a range of interventions to improve governance, science, and management; finance is an important enabler of a SOE, and underlies all ocean initiatives. The best ocean policies and practices can be undone by inadequate finance and by economic externalities that undermine conservation and sustainable use. The purpose of this perspective was to synthesize the current status and challenges in financing a SOE, and to outline actions to overcome the current barriers. Overall, the main barriers that prevent adequate financing of a SOE include a weak enabling environment for attracting sustainable ocean finance, insufficient public and private investment in the ocean economy, and the relatively high-risk profile of ocean economic sectors.

To turn the above challenges into opportunities, the public and private sectors need to create and better mobilize a full suite of financial tools and approaches, insurance, fiscal and market incentives, and strengthen key aspects of the enabling environment to support the transition to an ocean economy that is sustainable and inclusive by making the benefits it generates available to all, especially, women, youth, and marginalized communities. The most significant action will be to influence future mainstream finance. By providing clear principles, frameworks, guidance and metrics and proactively avoiding known illegal and harmful activities, substantial amounts of ocean financing would be redirected towards sustainable development pathways, creating long term and positive systemic change. If our recommendation to allocate a higher proportion of ocean GDP to the attainment of a SOE is followed by half of the world’s maritime countries, that alone could generate the seed money needed to incentivize the kind of public and private investments needed to ensure a SOE. The big message from this contribution is that a significant increase in sustainable ocean finance will be required to ensure a SOE that benefits all, including a broad section of society and businesses in developing as well as developed countries. In our opinion, the centrality of adequate finance to ensuring a SOE is such that the world may need a Paris Agreement (on climate change) type effort to meet the needs.
