# Author Correction: Financing a sustainable ocean economy

**DOI:** 10.1038/s41467-022-29418-x

**Published:** 2022-03-28

**Authors:** U. Rashid Sumaila, Melissa Walsh, Kelly Hoareau, Anthony Cox, Louise Teh, Patrízia Abdallah, Wisdom Akpalu, Zuzy Anna, Dominique Benzaken, Beatrice Crona, Timothy Fitzgerald, Louise Heaps, Ibrahim Issifu, Katia Karousakis, Glenn Marie Lange, Amanda Leland, Dana Miller, Karen Sack, Durreen Shahnaz, Torsten Thiele, Niels Vestergaard, Nobuyuki Yagi, Junjie Zhang

**Affiliations:** 1 Fisheries Economics Research Unit, University of British Columbia, Vancouver, BC Canada; 2 Marine Conservation Finance Consulting and Ocean Finance Initiative, Asian Development Bank, Metro Manila, Philippines; 3 University of Seychelles James Michel Blue Economy Research Institute, University of Seychelles, Anse Royale, Seychelles; 4 Organization for Economic Co-operation and Development, Paris, France; 5 Universidade Federal do Rio Grande—FURG, Rio Grande, Brazil; 6 Ghana Institute of Management and Public Administration, Accra, Ghana; 7 SDGs Center Universitas Padjadjaran, Kota Bandung, Indonesia; 8 Australian National Centre for Ocean Resources and Security, Wollongong, NSW Australia; 9 The Royal Swedish Academy of Science, Stockholm, Sweden; 10 Environmental Defense Fund, Washington, DC USA; 11 WWF-United Kingdom, Surrey, UK; 12 The World Bank, Washington, DC USA; 13 Oceana-Europe, London, UK; 14 Ocean Unite, Washington, DC USA; 15 Impact Investment Exchange (IIX), Singapore, Singapore; 16 London School of Economics, London, UK; 17 University of Southern Denmark, Odense, Denmark; 18 University of Tokyo, Tokyo, Japan; 19 Duke Kunshan University, Kunshan, China

**Keywords:** Environmental social sciences, Environmental economics

Correction to: *Nature Communications* 10.1038/s41467-021-23168-y, published online 08 June 2021.

The original version of this Article contained an error in Box 2, in which the Seychelles Conservation and Climate Adaptation Trust funding was written as (USD 75,000 in competitive grants per year). The correct value is USD 700,000. This has been corrected in both the PDF and HTML versions of the Article.

